# Mechanisms Driving Microbial Community Composition in Anaerobic Co-Digestion of Waste-Activated Sewage Sludge

**DOI:** 10.3390/bioengineering8120197

**Published:** 2021-11-30

**Authors:** Jan Torsten Jeske, Claudia Gallert

**Affiliations:** Department of Microbiology—Biotechnology, University of Applied Sciences Emden Leer, 26723 Emden, Germany; claudia.gallert@hs-emden-leer.de

**Keywords:** anaerobic co-digestion, semi-continuous microbial reactor, microbial community

## Abstract

Anaerobic co-digestion (Co-AD) is used to increase the effectiveness of anaerobic digestion (AD) using local “wastes”, adding economic and environmental benefits. Since system stability is of existential importance for the operation of wastewater treatment plants, thorough testing of potential co-substrates and their effects on the respective community and system performance is crucial for understanding and utilizing Co-AD to its best capacity. Food waste (FW) and canola lecithin (CL) were tested in mesophilic, lab-scale, semi-continuous reactors over a duration of 120 days with stepwise increased substrate addition. Key performance indicators (biogas, total/volatile solids, fatty acids) were monitored and combined with 16S-rRNA amplicon sequencing to assess the impact of co-substrate addition on reactor performance and microbial community composition (MCC). Additionally, the latter was then compared with natural shifts occurring in the wastewater treatment plant (WWTP, source) at the same time. An almost linear increase in biogas production with both co-substrates at an approximate 1:1 ratio with the organic loading rate (OLR) was observed. The MCCs in both experiments were mostly stable, but also prone to drift over time. The FW experiment MCC more closely resembled the original WWTP community and the observed shifts indicated high levels of functional redundancy. Exclusive to the CL co-substrate, a clear selection for a few operational taxonomic units (OTUs) was observed. There was little evidence for a persistent invasion and establishment of microorganisms from typical primary substrates into the stable resident community of the reactors, which is in line with earlier findings that suggested that the inoculum and history mostly define the MCC. However, external factors may still tip the scales in favor of a few r-strategists (e.g., *Prolixibacter*) in an environment that otherwise favors K-strategists, which may in fact also be recruited from the primary substrate (*Trichococcus*). In our study, specialization and diversity loss were also observed in response to the addition of the highly specialized CL, which in turn, may have adverse effects on the system’s stability and reduced resilience and recovery.

## 1. Introduction

Anaerobic digestion (AD) is used in wastewater treatment worldwide, mainly to reduce and stabilize waste sludge that accumulates during the treatment process [1]. Through the combustion of the produced biogas (mostly CH_4_ and CO_2_) in on-site combined heat and power plants (CHPs), AD also allows for a significant reduction in the running costs and helps to minimize the environmental impact. Biogas is produced via microbial metabolism, by which organic matter is degraded in a series of sequential and parallel steps by *Bacteria* and *Archaea* in a trophic cascade [2]. The primary and waste-activated sewage sludge (PWASS) that is most commonly used in AD in wastewater treatment plants (WWTPs) is typically energy depleted and highly metabolized so that AD reactors are often running well below their potential production capabilities. This unused potential may be harnessed by anaerobic co-digestion (Co-AD) with an energy-rich co-substrate alongside the primary- and waste-activated sewage sludge (PWASS) [3,4,5,6]. Biogas production is still considered an emerging technology for renewable energy production worldwide, with many countries relying on its implication to achieve climate goals, such as China’s pledge to achieve CO_2_ neutrality by 2060 [7] or the European Commission’s aim to reduce greenhouse gas emissions by 55% by 2030 [8]. Several ecological challenges for a green economy could be addressed using Co-AD. Especially in wastewater treatment, co-digestion could play a major role in achieving climate goals without neglecting or interfering with a plant’s primary role. Biogas production might be tripled when solid organic waste is loaded in concentrations of up to 94% of the sludge organic loading rate (OLR) [4] and methane yields might be improved significantly, for example, from 138 mL CH_4_ g^−1^ total solids (TS) in single digestion of sewage sludge to as much as 294 mL CH_4_ g^−1^ TS from the Co-AD of sewage sludge with food waste (FW) and swine waste [3]. Yet not all co-substrates are alike, and while using fat, oil, and grease (FOG) as co-substrates will lead to high biogas and methane yields, it also leads to operational problems, such as foaming or the accumulation of volatile fatty acids (VFA), which in turn, may have inhibitory effects on the AD [9]. Other co-substrates might lead to the acidification or accumulation of ammonia or heavy metals, leading to performance issues or even a reactor failure [10,11]. Availability and a lack of competition is another key factor in choosing a co-substrate for municipal WWTPs, as, ideally, a local waste product is used. Co-substrate specific effects on the reactor community should be evaluated with care and tested on a case-by-case basis, as was done for the co-digestion of algae and other organic wastes, which resulted in very different methane yields. It was shown that each co-substrate came with its own challenges, such as organic overloading, elevated sulfur- or phosphorous concentrations, or induction of changes to the VS and chemical oxygen demand (COD) in the digested sludge [6]. Food wastes, although generally a beneficial co-substrate, often come with their own regional and culture-specific challenges and limitations. For example, Chinese FWs, exemplified via a proxy in this study, typically have high salt and oil content, which may interfere with or change key players in the AD process [12,13].

Productivity of the AD process is also tightly linked to the synergistic activity of complex microbial communities, and sequencing-based analysis of the microbial community composition (MCC) has been proven to be crucial in linking adaptation and changes in the MCC to changes in environmental conditions and function of the AD [14,15]. While the AD process is typically robust, it relies on the stability of the reactor community and its relative composition [1,15,16,17]. The core microbiome of AD microbial communities has been shown to be rather small and remarkably similar, often consisting of a majority of K-strategists [18], with as few as 300 OTUs representing up to 70–80% of the core community, and with a prevalence of over 80% even between different AD systems [16,19]. However, while there are considerable overlaps between similar systems, distinct core microbiomes have been demonstrated based on the respective feeding stocks and origins, for example, WWTPs that have distinct core communities in opposition to AD reactors that treat agricultural and bio-wastes [19]. While process performance and functionality are linked to the MCC [20], it has also been demonstrated that dynamic changes in microbial community composition are possible while retaining process performance and capability [21,22], which in turn, indicates plasticity and functional redundancy [23]. Understanding the mechanisms that shape and influence MCC in engineered AD ecosystems could lead to better control and new mechanisms of regulating the AD process performance and stability.

A key component in shaping the microbial community is the source, that is, active biomass of inflow or input in natural systems, or inoculum in laboratory settings. Inoculation with an active microbial community may mitigate risks of process failure during start-up or facilitate recovery after process disturbances [24]. Opposing—or, at least, conflicting—views exist on how the MCC is thereafter shaped over time. On one hand, it has been proposed that the process is deterministic, and with a given set of environmental conditions, one certain community would form. This, in turn, would allow for somewhat direct control on the MCC from an engineering perspective, via setting and regulating external factors, such as the temperature or hydraulic/solids retention time (HRT/SRT) alone, as was demonstrated on simplified AD models with consistent and well defined low complexity media, and without an influx of MCC [17]. Contrasting ecological models proclaim that a drift in the MCC is to be expected, and even the same starting community with the same conditions might lead to differing MCC outcomes [25] alongside a potential loss of ecosystem functions [26]. MCCs and functional parameters of AD and Co-AD have mostly been studied previously in short-term batch experiments [27], which are well suited for understanding the framework of these two concepts. However, the lack of dynamic input does not allow for also adequately addressing a third mechanism that, only recently, has gained major attention—potential active microbial invasion from the respective sources, which might be the key driver in shaping the microbial community and structure [28,29,30,31]. Semi-continuous or continuous lab-scale reactors would much better allow for investigating such a mechanism, but they come with a series of operational challenges and considerable trade-offs in terms of feasibility versus simulation. Initial data on invasion in AD indicates that the factual influence is low and only a small percentage of microorganisms may establish within the AD system [30]; however, major research gaps and numerous unknowns persist that have yet to be disclosed [30].

The aim of this work was to identify changes in MCCs and biogas and methane production in a system as close to a full-scale reactor as possible while still maintaining the control that a laboratory-style reactor experiment provides. As the feedstock may define the community development, as proposed in [17], “overfeeding” was avoided to better represent the true operating conditions. Additionally, this approach also avoids artificial changes in the MCC from a typical WWTP AD towards that of a biogas plant-like digester [19]. Therefore, the MCC, and the processes that would occur under normal operational conditions in a WWTP, which attempts to implement Co-AD, were assessed. Additionally, changes that might only be established over extended time periods, such as microbial invasion or even deterministic or statistic effects that could lead to MCC shifts and, thus, changes in the process performance, were recorded.

## 2. Materials and Methods

### 2.1. Sampling and Experimental Setup

A total of 6 reactors, with a 1 L reactor volume and a 500 mL continuously stirred working volume each, were operated over a period of 120 days, during which the temperature was kept constant at 37 °C. The inoculum was obtained from an anaerobic digester located at a midsized WWTP in northern Germany, which is sized for 90,000 inhabitant equivalents IE (72,000 IE working capacity) with a factual workload of sewage of at 3.97 × 10^6^ m^3^ a^−1^. The sewage is treated via the mechanical retention of large solids, the separation of sand and grit in aerated chambers, and the settling of suspended solids in primary settling tanks. The subsequent biological removal of nutrients is achieved via two intermittingly run activated sludge units, (4.398 m^3^ each, with simultaneous chemical phosphorus precipitation), succeeded by secondary clarifiers (2 × 2.922 m^3^). Primary (PS) and waste-activated sewage sludge (WAS) are combined (PWASS, 60:40), and after pre-thickening, the raw sewage sludge is fed to two digesters (2000 m^3^ volume each) for AD. For the experiments, PWASS was collected monthly, directly upstream of the digesters, and portioned in one-day-feed doses. The resulting eight batches of PWASS were used successively over both experiments as the main substrate. The digested sludge was taken on 28 October 2019 for Experiment 1 (FW). In the second experiment, canola lecithin (CL) was used as a co-substrate, and the respective inoculum for the CL experiment was obtained on 4 June 2020. The main chemical compositions of the primary substrate PWASS and the co-substrates FW and CL are shown in Table 1. In the FW experiment, 25 mL of content from the control reactor (*n* = 3) was replaced with 25 mL of PWASS every 24 h, excluding weekends, leading to an HRT-stabilized system with an HRT of 20 days during the week. The experimental reactors (*n* = 3) were treated similarly, but, following a one-month feeding period without co-substrate, they were supplemented with co-substrate as an added percentage of the VS of the respective raw sludge batch (Table A1). The CL experiment started immediately after a restart with fresh inoculum. The amount of co-substrate was determined as a percentage of the VS and was increased stepwise over the duration of the experiment from 0 to 30% (Table A1). To counteract the acidification that occurred during the feeding period with PWASS batch No. 6, the pH was raised to 7.2 with NaOH to stabilize the reactors. The co-substrate for the FW experiment was obtained from a Chinese restaurant. Food and kitchen wastes were collected over one day, after which they were collected, mushed, and frozen at −20 °C. For the second co-substrate, an industrial by-product was used, specifically, a mixture consisting of canola lecithin and small amounts of citrate, which is used to waterproof food paper/parchment and accrues in local industries.

### 2.2. Analytical Procedures

The methane production was monitored online with methane sensors (BlueSens, Herden, Germany), and the produced gas volume was recorded with MilliGascounter^®^ (Ritter, Germany). Volatile fatty acids (VFA) were measured weekly via gas chromatography according to the method described by Uhlenhut et al. [32], and the pH was measured offline in the reactor excess daily during feeding (Hanna Instruments, Vöhringen, Germany). The TS and VS were measured by gravimetric analysis according to the Standard Methods 2540B and 2540E, respectively [33]. The chemical oxygen demand (COD) and total nitrogen of all substrates were analyzed with a cuvette test from Hach Lange, LCK338 and LCK514, according to the manufacturer’s instructions. The Weender analysis technique of the substrates was performed by LUFA Nord-West, Institut für Futtermittel, Oldenburg, Germany, according to their standard procedures.

### 2.3. DNA Extraction and 16S rRNA Gene Amplicon Sequencing

Samples for DNA extraction were taken weekly from the lab-scale reactors and stored at −20 °C. DNA for amplicon sequencing was extracted using the AllPrep PowerViral DNA/RNA kit (Qiagen, Düsseldorf, Germany) and the Soil DNA Isolation Plus kit (Norgen, Thorold, ON, Canada) in triplicates, according to the manufacturers’ instructions. The hypervariable V3–V4 region of the 16S rRNA gene was used as a marker to analyze the microbial community composition (MCC) in the lab-scale reactors and the mesophilic full-scale WWTP anaerobic digestor. Sequencing of the barcoded amplicons was performed using the Illumina MiSeq platform with V2 chemistry (Illumina Inc., San Diego, CA, USA) according to the manufacturer’s instructions. Primers Pro341f/Pro805r [34] targeting the domains *Archaea* and *Bacteria*, respectively, were used to generate barcoded amplicons for sequencing. Libraries were prepared using the NexteraXT kit (Illumina Inc., San Diego, CA, USA).

### 2.4. Data Handling and Analysis

Sequence processing was performed according to the method of Dyksma et al. [35]. In summary, raw reads were filtered and low-quality reads were removed. Paired-end reads were then assembled, trimmed, and denoised. Then, the chimeras were removed and the reads were aligned to the SILVA database v132. The sequences were then clustered into operational taxonomic units (OTUs) at a 97% similarity level and classified using the sina-classifier. After quality filtration, a total of 12,147,396 reads were obtained from 64 samples (Table S1). Sequence analysis was done with R, using the packages phyloseq, vegan, and microbiome. The influence of the experimental treatments and factors on biogas and CH_4_ production was tested using linear mixed-effects models using the lme4 package. Factors that were included in the analysis, both as fixed and/or random effects, were OLR and co-substrate, weekday, individual reactor, PWASS batch, pH, TS, VS, and runtime, with the final model using OLR and co-substrate as fixed factors, and weekday and reactor as random effects that were nested within the PWASS batch. The influence of the experimental treatments and known environmental factors on the MCC were tested for significant influence with Permanova and PCoA based on the Bray–Curtis dissimilarity matrix at the OTU level. Only factors that significantly influenced the CL-MCC were used in the canonical analysis of the principal coordinates (CAP). The same factors that were found in the CL experiment were used for the FW experiment. Additionally, same-day sampling of biological replicates versus temporally separated sampling of the same reactor was used as a proxy for internal variation in the FW analysis and ordination. Graphical presentations were made using the package ggplot2.

## 3. Results

### 3.1. Substrate Characterization, Biogas Production, and Reactor Performance

The main parameters of the different substrates primary and waste-activated sewage sludge (PWASS), food waste (FW), and canola lecithin (CL) were analyzed and the results are summarized in Table 1. The measured parameters for the sampled PWASS that were used as the primary substrate were all within the normal parameters that were reported from the source plant. The TS and VS used in this study were slightly below the mean values reported from the WWTP, although they had a considerably lower standard deviation and variation.

The first co-substrate (FW) was derived from a Chinese restaurant (typical composition) and after homogenization, the dry matter content was 221.3 g L^−1^, of which 89.33% were volatile solids (VS). The COD of 256.7 g L^−1^ and the total N of 4.2 g L^−1^ are approximately 10 and 7 times higher, respectively, than those of the PWASS. The second co-substrate (CL) had a total solids content of 464.1 g L^−1^, with 99.84% being VS, resulting in a high COD concentration of 529 g L^−1^—more than twice that of the FW, however, with only 1/15th of the total N content in comparison (Table 1).

**Figure 1:**
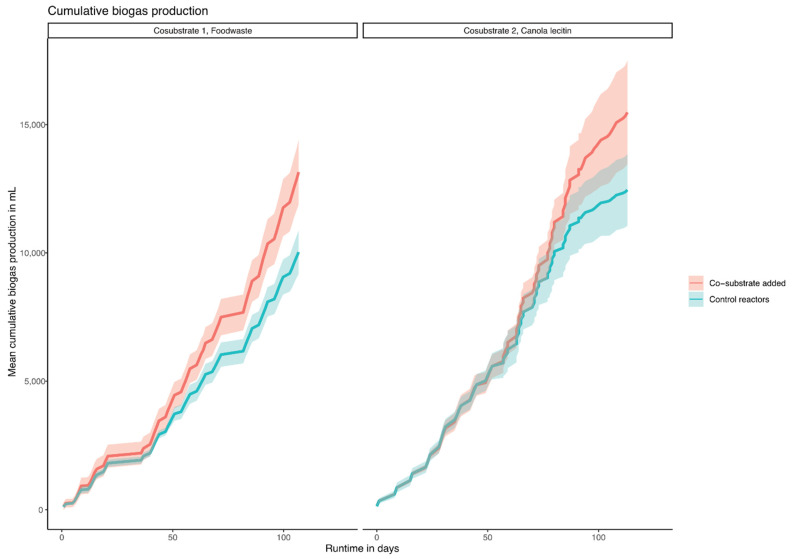
Cumulative biogas production of the two experiments per reactor, in mL, with standard deviation, over time. Food waste on the left, canola lecithin on the right.

In order to mimic the operational conditions of the WWTP, the mesophilic, semi-continuous, lab-scale reactors were operated with the same HRT (days) and OLR (g VS m^−3^ and day) as the full-scale WWTP, and the respective co-substrates were added as a percentage of the OLR of the main substrate PWASS (Table A1). The cumulative biogas production with the main substrate (PWASS) and the co-digestion with the additional FW and CL substrates are shown in Figure 1. With increasing amounts of co-substrate, the total amount of biogas increased. The addition of the co-substrate was calculated according to the VS content of the main substrate PWASS, which varied with the operational conditions of the WWTP and VS concentrations ranging from 16.1 to 27.6 g L^−1^ (Table A1). The OLR in the experimental reactors, thus, varied with the OLR of the control groups (0%). The specific biogas yield (ml g VS^−1^ added) in the FW experiment was 306 ± 67 (OLR 1.03 kg VS m^−3^ d^−1^, 0%) and increased to 378 ± 67 after adding FW (OLR 1.13 kg VS m^−3^ d^−1^, 10%).

With increasing amounts of added co-substrate, the total amount of biogas, as well as the specific biogas yield, increased proportionally. The average methane content of the biogas was 67.4%. The addition of FW to the experimental reactors resulted in increased biogas production, with little effect on the specific biogas production (10% FW resulted in 16% more biogas, 20% FW in 30%, and 30% FW in 41% more biogas). In the experiments with CL as a co-substrate, the specific biogas yield was observed to be 299 ± 99 mL g VS^−1^ added (OLR 1.13 kg VS m^−3^ d^−1^, 0%) with a methane content of 65.7%, indicating a lower oil content compared to that proposed for similar substrates, such as rapeseed cakes with a 15% oil content [36]. As can be seen in Figure 1, the cumulative biogas production increased after an adaptation phase and accumulated to 6.65 L of biogas in the control reactors and 10.57 L of biogas with 30% co-substrate during the last feeding period (Figure 1).

### 3.2. Microbial Community Composition

#### 3.2.1. Archaea

**Figure 2:**
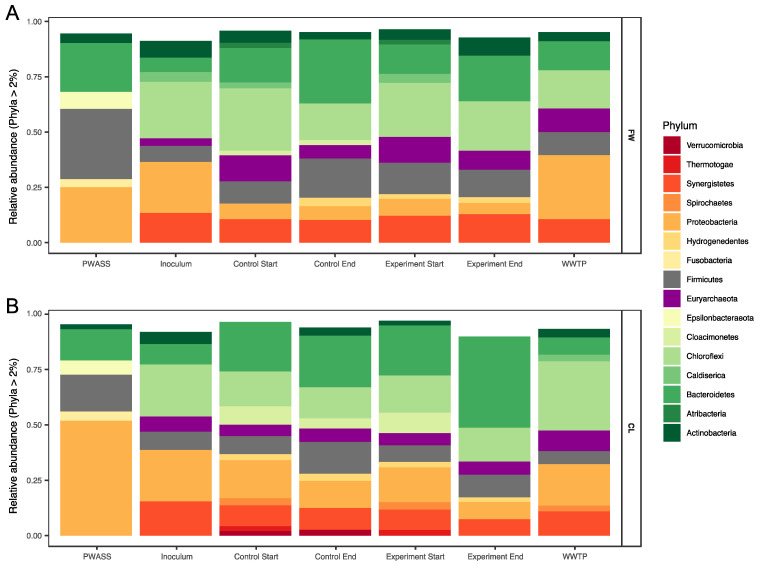
Microbial populations and community composition changes with time and treatment. Phylum-level bar-chart representation of the major taxa (>2% of the total MCC) of the two experiments ((**A**) = Food waste, (**B**) = Canola lecithin). A representative raw sludge sample is shown on the very left of each bar plot, followed by the inoculum of the lab-scale reactors on the day of sampling (second from left) and the starting community after 4 weeks in the controls and experimental reactors (3rd, and 4th from left, respectively) and the MCC at the end of the sampling period (3rd last and 2nd last from the left). The last column represents the MCC of the full-scale WWTP AD at a timepoint corresponding to the end of the respective experiment.

The relative abundance of amplicon sequences was used to characterize the microbial community composition (MCC) in the reactors and investigate differences between the treatments, stability of the communities, and changes in the MCC as a response to the changing environmental conditions, that is, changes caused by the PWASS batches or the added co-substrate and compare those with the natural changes occurring in the WWTP AD. Archaeal sequences initially comprised 3.5 and 6.75% of the relative abundance of the total microbial community in the two inocula (FW and CL, respectively, Figure 2), which stabilized at ~11.1 ± 1.4% in the six FW experiment reactors after the first 4 weeks, in which no co-substrate was added. The archaeal population remained largely stable within the FW experiment, with a slight decrease in the relative abundance towards the end of the experiments (8.5% and 6.6% of the total MCC in the experimental (co-substrate-fed) and control reactors after 125 days, respectively). The archaeal community in the CL was less stable overall and the relative abundance of the archaeal sequences was reduced to as little as 3.6% around day 71 in the experimental reactors and 4.3% in the control reactors from an original 5.6 ± 0.5% in the six CL reactors 4 weeks after inoculation. However, all CL reactors (co-substrate-fed and control reactors) recovered to 5.8% (experimental) and 6.1% (control) after 92 days. In comparison, the average relative abundance of archaeal sequences in the full-scale WWTP was at 7.2 ± 1.3%, with one major outlier at 4.9% over the duration of both experiments.

**Figure 3:**
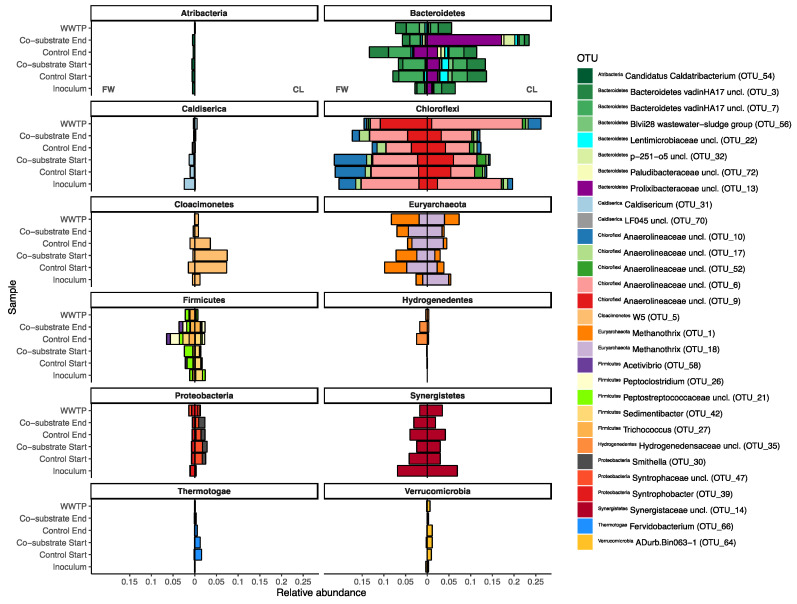
Divergence bar plot showing samples from the inocula, the lab-scale reactors, and the WWTP. Plotted are the OTUs with the top 30 Permanova coefficients identified as the response variables when “Experiment” was chosen as the independent categorical factor and MCC as the response variable (see also Figure 4). Shown are the OTUs that have either the most positive (15, max = 0.017) or most negative (15, min = −0.017) coefficients, thus most differentiating the two MCCs from each other. Bars are plotted phylum-wise, with bars extending from the centerline to the left representing the relative abundance of the respective OTU in the FW experiment, and bars to the right representing the CL experiment. The WWTP sample at the top of each plot represents a WWTP sample at the end of the respective experiment, that is, approximately 100 days past the inoculum.

The archaeal community composition (ACC) in all reactors and with both co-substrates was dominated by OTUs classified as members of the genus *Methanothrix*, with three OTUs contributing 83% of the ACC in the inoculum of the FW reactors, and the same three plus a fourth OTU contributing a total of 87% of the ACC in the inoculum of the CL reactors (Figure 3). Yet, the relative abundances and ratios between them differed considerably (*p* < 0.01), and while the FW inoculum was split almost in half between the two most dominant OTUs, 1 and 18 (42 and 31% of the ACC, respectively), the CL inoculum was heavily dominated by OTU 18, contributing a majority of 75%. These two dominant *Methanothrix* OTUs remained almost at a stable ratio towards each other throughout the FW experiment. Similarly, both treatments (control, without, and experimental, with co-substrate) in the CL experiment exhibited a similar trend towards a more equalized ratio between the two dominant *Methanothrix* OTUs. Moreover, at the end of the equilibration phase, and exclusive to the CL experiment, some OTUs were detected in considerable abundances (>5% relative abundance) that were identified as members of the Genus *Methanolinea*, *Methanospirillum*, and Candidatus *Methanofastidiosum*, which were virtually absent in the FW experiment and in the inocula of both experiments, respectively. All three of them, as well as the ACC as a whole, appeared unaffected by the co-substrate addition (*p* < 0.05).

**Figure 4:**
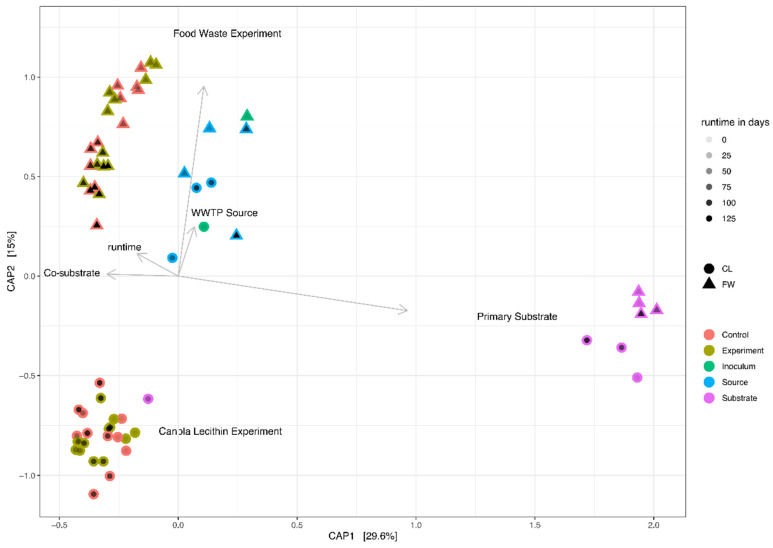
Constrained ordination plot (CAP) of the MCC of the two experiments, with environmental factors added. The ordination is a combination of shape and color to represent the operational treatment and the corresponding PWASS batch simultaneously, as a third dimension. MCC from both experiments, food waste (FW, triangles) and canola lecithin (CL, circles), are shown with environmental predictors, identified as significant in the Permanova on top of the ordination. Runtime is added to each point with alpha shading from light to dark, black symbols being the maximum runtime during each experiment.

Only minor overlaps of the (Co-)AD ACC with the PWASS ACC were found, although the relative abundance of the archaeal sequences was already considerably lower in the PWASS (typically <0.5%). Similarly, the ACC (and BCC) of the full-scale WWTP AD was observed to be much more similar to the community composition of the FW experiment, while also remaining significantly different (*p* < 0.05) from either of the two experimental communities (Figure 4). Additionally, a minor decline in the Shannon diversity was observed, from 2.1 ± 0.2 in the first half of the CL experiment to 1.9 ± 0.3 in the second half, which, however, was pronounced much more drastically among the bacterial taxa (see Section 3.2.2).

#### 3.2.2. Bacteria

The most abundant phyla detected in the FW inoculum were *Chloroflexi* (25.2%), *Proteobacteria* (23.2%), and *Synergistetes* (13.1%) with *Actinobacteria*, *Firmicutes*, *Bacteroidetes*, and *Caldiserica* contributing the majority of the remaining community assemblage (7.9, 7.2, 6.9, and 4.3%, respectively). This was very similar to the community composition of the CL inoculum, in which *Chloroflexi*, *Proteobacteria*, and *Synergistetes* made up the bulk of the community as well (23.5, 23.4, and 15.7%, respectively), with *Bacteroidetes* and *Firmicutes* in almost equal amounts (8.8% and 8.4%, respectively), and while *Actinobacteria* appeared to have a slightly reduced relative abundance (5.6%, Figure 2A,B).

During the equilibration phase, some major shifts were observed in both experiments. Most prominently, both experiments experienced a surprisingly similar large drop in the relative abundance of *Proteobacteria* (~16%, from ~23% to ~7% in both experiments). In the FW experiment, the resulting gap was mostly filled by *Bacteroidetes* (and *Euryarchaeota,* see Section 3.2.1), with an increase in the relative abundance to ~15% (and 12%, respectively), and subsequent minor reorganization in the remaining phyla (Figure 2). Similar patterns were observed for *Bacteroidetes* in the CL experiment (with no comparable rise in the euryarchaeal abundances, however). In the CL experiment, a number of other phyla emerged, namely *Cloacimonetes*, *Spirochaetes*, and some *Thermotogae*. Additionally, and in opposition to the FW experiment, *Synergistetes* also experienced a decline in relative abundance. Some of these shifts can even very prominently be observed at OTU levels (Figure 3), where large differences between the two experiments can already be observed, despite not yet being treated differently. For example, a majority of the shifting *Bacteroidetes* in the FW experiment were assigned as *Bacteroidetes* vadinHA17, which did not show a similar increase in the CL experiment. In the latter, especially unclassified *Lentimicrobiaceae* and *Prolixibacteraceae*, as well as *Paludibacteraceae* were observed to have higher relative abundances (Figure 3, *Bacteroidetes*). Interestingly, while no major shifts at the phylum level were observed between the experiments for *Chloroflexi*, which, in large part, were made up of OTUs assigned as different unclassified *Anaerolineaeceae*, discrete shifts among those were observed that were specific for either of the two experiments but showed no specific correlation with the co-substrate addition. The same was not true for the earlier described *Bacteroidetes*, where especially the unclassified *Prolixibactaeraceae* (OTU 13) showed a significant (*p* < 0.01) positive correlation with the co-substrate addition (Figure 3, *Bacteroidetes*). Furthermore, in the late phase, the CL experimental reactors also saw an increase of members of the genus *Trichococcus* (OTU 27) from below 0.2% of relative abundance in the inoculum to between 1.3 and 1.9% in the experimental reactors, which was not observed in the control reactors. The same experimental reactors also experienced a parallel decline in the relative abundance of one of the core community members (*Bacteroidetes* vadinHA17). Similarly, *Fervidobacterium* and *Cloacimonetes* W5, which were previously described to often co-occur [37], appeared to be equally negatively impacted by the CL co-substrate addition. However, they were generally less abundant throughout the FW experiment and showed no correlation with the FW co-substrate addition (Figure 3, *Cloacimonetes/Thermotogae*). The two experiments could furthermore be differentiated by *Caldiserica*, which were much more prominent in the FW experiment, and *Verrucomicrobia*, which, in opposition, appeared mostly in the CL experiment. Another clear difference between the two experiments was observed among the *Proteobacteria*, where *Syntrophobacter* was observed to be specific for the FW experiment, while it was missing in the CL experiment, in which unclassified *Syntrophaceae* and *Smithella* were found instead, neither exhibiting any drastic response to co-substrate addition. As described above for *Archaea*, the WWTP AD community was distinct from the experiment BCC as well, with their respective internal shifts. Overall, the WWTP AD MCC was more similar to the FW MCC, yet with separate unique shifts throughout the duration of the experiment (Figure 2, Figure 3 and Figure 4). Especially higher relative abundances of *Proteobacteria*, much less plasticity among the *Bacteroidetes*, and *Chloroflexi*, but similar overall levels of change among *Euryarchaeota* as a representative of the ACC, and even more change fluctuation among members of the phylum *Synergistetes* were recorded.

Statistical analysis and Permanova revealed no significant influence of the co-substrate addition on the MCC in the FW experiment but did reveal a significant relationship between the MCC and runtime, irrespective of the treatment. No significant difference between the inocula and the WWTP AD was detected, however, and similar to the FW, a weak significant difference was detected based on the runtime, that is, a change in the community composition over time and within a system. Only within the CL experiment did statistical analysis reveal a significant (*p* < 0.01) correlation with the increasing co-substrate addition (Figure 4). Similarly, diversity remained at an even level throughout the entire FW experiment of 120 days, as represented by the Shannon index, which was at 5.37 in the inoculum (Table S2), and showed little variation over the investigated time-points, measured at 5.17 and 5.10 in the controls and co-digestion reactors, respectively, and at 5.43 in the WWTP AD after the same time span after sampling. The MCC in the CL experiment started with a similar level of diversity (Shannon index = 5.12), which remained mostly stable (5.10 to 5.25) in the controls throughout the experiment, while in the co-substrate-fed experimental reactors, the Shannon diversity decreased to as little as 4.67 with 30% co-substrate addition. At the same time, the WWTP AD MCC was found to be at 5.01 after the same ~100 days. The diversity in the primary substrate (PWASS) was generally higher, with very little overlap with the AD systems, a distinct, smaller core community, and more overall plasticity and variability. Here, the Shannon diversity was found to be at 5.98 ± 0.48 with one major outlier that also, in its community composition, resembled an AD MCC more than a typical PWASS MCC. The majority of the PWASS BCC was made up of *Proteobacteria*, which were often strongly reduced in the AD experimental and control reactors. As is typical for PWASS (Figure 2A,B, far left), members of the phylum *Firmicutes* typically represented the second largest group, among them members of the genus *Trichococcus* (OTU 27, between 2 and 4% of the total community), while the third most abundant phylum were *Bacteroidetes*.

## 4. Discussion

### 4.1. Substrates Characterization, Biogas Production, and Reactor Performance

The PWASS mixture had a typical composition of sludge derived from a mid-sized WWTP [38], and their respective TS and VS concentrations well represented the values for TS and VS that were reported from the WWTP during the same time frame and within the standard deviation of those values, although with considerably less variation (Table 1 and Table A1). The operational parameters that were used in the experiments were chosen in an attempt to mimic the WWTPs’ hydraulic retention time (HRT) while being applicable to a laboratory setting. Accordingly, a discontinued feeding during weekends was used, which resulted in slightly higher loading during weekdays and a period of extended fermentation during the weekends. As a result, characteristic sawblade-like biogas-production patterns (not shown) were observed, as also reported from similar studies [39,40], which differ from patterns of continuous biogas production at maximum capacity. This, in turn, leads to increased variability and higher standard deviation of the daily biogas production than what batch or large-scale continuously fed reactors would show. Therefore, average specific biogas production in the lab-scale reactors was slightly below the assumed specific yields obtained from the WWTPs—for example, 350 to 450 mL g VS^−1^ added when feeding the PWASS [41], or the 402 mL biogas g^−1^ VS in lab-scale reactors, reported by Aichinger et al. [4]—but well within the levels that have been reported from similar studies [39,40,42]. The differing OLRs during the different stages of experimental treatments (with 10%, 20%, and 30% co-substrate addition, Table A1) were a result of the sequence (number), and the variances of TS and VS of the PWASS batches used. These variances would vary on a day-to-day basis in a real-life plant and, thus, have no strong influence there, unlike in the experimental setup, where single batches were fed repetitively over extended periods. Such an approach has its own drawbacks, as inhibitors that might be found sporadically during the normal operation of a WWTP could, by chance, be much more highly concentrated in a single feeding batch and would accumulate during repetitive feeding. Such inhibitors could include undetermined iron sulfates and sulfides [11] that compete with methanogenesis when available in excess [43], or the Al_2_(SO_4_)_3_ that is used in the upstream biological unit of the corresponding WWTP to control filamentous bacteria [44]. However, the chosen method of feeding would, by design, also benefit and overemphasize invasion patterns from the PWASS to the AD, especially in the sense of establishment linked to mass effects [29,30,45]; determining the degree to which this happens was one of the main aims of this work.

The second main focus of this study was to assess the influence of the addition of a co-substrate on biogas production and microbial community composition and stability, based on waste products that could be acquired locally but would also be available in countries where anaerobic co-digestion or even anaerobic digestion of PWASS is not yet established, such as China, Vietnam, Indonesia, and many other countries worldwide. As a compromise, two distinct waste products were chosen—one representing a complex mixture of different organic polymers typical of a food-derived waste (FW) and collected from a restaurant (e.g., from a Chinese restaurant). The second co-substrate (CL) was representative of few different organic polymers that accrue during a specialized industrial production process, with less complexity and more defined in terms of its relative composition. Both co-substrates were high in organic content and a high degree of digestibility can be assumed for both [6], but specific rates were not confirmed experimentally with the specific substrates. Food waste, as well as other organic municipal wastes, have previously been described as very beneficiary co-substrates that can enhance the specific methane and biogas production in WWTP AD [3,5]. Björn et al. [5], for example, observed a 1.14-fold increase in specific biogas production when tested with the addition of up to 233% FW of the sludge OLR. In the experiments described herein, the co-substrate dosages were within the possible range, allowing normal plant operation. The addition of FW led to an almost linear correlated increase in biogas production, while slightly different patterns were observed with the addition of CL. The DWA M 380 datasheet on Co-AD [36] proposes a specific biogas yield of 615 mL g VS^−1^ for biowastes as the only substrate, corresponding to an increase of 61.5 mL g VS^−1^ after adding 10% of such a substrate, which is in good accordance with the measured values.

During the co-digestion of CL—which is a yellow-brownish fatty, amphiphilic substance and, typically, a mixture of glycerophospholipids—a delayed increase in biogas production was observed (Figure 1). Moreover, almost no additional biogas production was observed in the very early phase (10%), which might be explained by the ongoing adaptation of the microbial community to this “unusual” substrate (see below) that negatively affected the biogas production or additional adverse effects caused by an earlier PWASS batch, as stated above. Ultimately though, this trend was reversed with higher dosages of the co-substrate, resulting in higher specific production rates compared to the controls towards the end of the experiment. This “specific” increase could be related to the selection and specialization of the microbial community as a direct response to the co-substrate addition.

### 4.2. Microbial Community Composition

The ACC, in general, remained stable throughout the co-digestion with FW and did not exhibit any significant response to the addition of the co-substrate or the PWASS batches, similar to what has been reported in other studies [19,46,47]. Similar behavior has previously been described in full-scale systems [19,48,49]. In addition, the methanogenic community observed in the experiments and the full-scale WWTP resembles a typical AD-reactor ACC [49,50]. The two dominant OTUs (1 and 18) that were present in approximately equal abundances in the FW inoculum, with a stable ratio during the early phases, experienced quite drastic shifts in the relative abundance of only one of the OTUs towards the later stages (Figure 3). OTU 1 was already much less abundant at the beginning of the CL experiment but also experienced a similar decline towards the later stages of the experiment. This was not observed in the full-scale WWTP. Unfortunately, no metabolomic or transcriptomic data is available, but a correlation with the runtime, that is, a proxy for the history of the community since inoculation, was found to be significant (*p* < 0.01, Figure 4). Additionally, relative abundances were too high, initially, to exclusively assume dilution effects. Thus, a negative selection linked to the laboratory conditions, r- and K-strategists, with OTU 1 being the less resilient r-strategist [51], or a reaction to general adverse effects—which would have to be further investigated—seem possible but remain hypothetical at this point. Similar diversity and specialization in different AD systems have recently been described with, for example, *Methanothrix concilii* and other methanogenic *Archaea*, allowing for an assumption to be made for a considerable range of genome plasticity among them [52]. Furthermore, a shift towards hydrogenotrophic *Archaea* in the CL experiment was observed, where, in combination with the somewhat lower specific biogas production, an unknown stressor could be assumed. Previous research has shown that although hydrogenotrophy is energetically more favorable, per se, it is often outcompeted by acetoclastic methanogenesis [53]. The observed increase in hydrogenotrophic methanogenesis might be a reaction to the shifting pH of unknown cause that was observed during the runtime of the experiment, and while acetoclastic methanogens have been shown to have their growth maxima around a neutral to slightly alkalic pH [54,55], hydrogenotrophic *Archaea* seem to be favored under more extreme conditions in either direction [54,56,57], and species-sorting, neutral, and mass effects could be at play [25,58,59,60].

With acetoclastic methanogenic *Archaea* being at the bottom end of the anaerobic trophic cascade [61], the least amount of change in their relative abundance or contribution to changes in the MCC was expected—at least, solely as a response to the co-digestion and assuming that the co-substrate addition would not lead to massive imbalances or the accumulation of inhibitory substances or VFAs, neither of which was to be expected or observed under the given experimental conditions. Shifts to the relative abundance of bacteria involved in the primary metabolization and decomposition of complex organic polymers, on the other hand, are likely to be observed as a selective response to co-substrate addition. Yet, because of the high levels of functional redundancy and frequent and widespread internal variability typically found among the BCC [15,17,62], plus the typically high compositional complexity of the substrates, changes are much less likely to be detected and might occur in less abundant but still important key organisms that might easily be overlooked.

To untangle and unravel these shifts, first, the core bacterial community was identified, that is, members of the MCC with a high likelihood of fulfilling a specific niche within the community were pulled from the amplicon data via detection and prevalence thresholds. The thus-identified OTUs were then screened for those contributing most towards differentiating the respective controls from one another, that is, the OTUs that were likely selected by neutral effects after inoculation. Subsequently, these OTUs of interest (OOI) were examined with all treatments, the PWASS, and the full-scale WWTP AD. Overall, the core bacterial community was strikingly similar in both experiments and the WWTP AD. Most OTUs comprising the core community were identified as members of the families *Anaerolinaceaea*, *Synergistaceae*, and *Bacteroidetes*—although with differing relative abundances of the respective OTUs between experiments and treatments. The remainder of the core community itself mostly resembles a typical AD BCC, as has been described in previous research, although with lower numbers of *Proteobacteria* especially [19,52,63].

Apart from a few exceptions in the CL experiment, little to no overlap of the core community or the identified OOIs with the PWASS was detected. This indicates that there were likely no invasion or establishment mechanisms [29,30] from the PWASS to the reactor community, which, in turn, suggests that the latter were primarily defined by their own respective history and the respective inoculum MCC [25]. This is exemplified in Figure 4, where there are four separate clusters visible, each belonging to a single environmental system (FW or CL experiment, WWTP AD, PWASS), with practically no overlap of the systems with each other (except one PWASS batch that might be identified as “problematic”, caused by WWTP operational procedures and personal communication), and less variation between the treatments within one experiment than amongst the experiments and the respective WWTP AD (including the two inocula). Especially among the *Anaerolinaceae*, multiple OTUs with very distinct, experiment- but not treatment-related abundance patterns (Figure 4, *Chloroflexi*) were observed. However, since all of these *Anaerolinaceae* could only be assigned as unclassified to anything below the family level, further inference would be purely speculative at this point. Similar patterns suggesting history/neutral mechanisms at play were observed for representatives of all the other major phyla as well (Figure 4).

Unlike the individual, inoculum-related, and neutral theory-correlated drift patterns visible in the WWTP AD, the FW experiment, and the CL experiment controls, the co-substrate-fed reactors in the CL experiment exhibited patterns indicative of species sorting and selection, and likely even invasion or recruitment from the primary substrate (OTU 27). This might be a direct response to the somewhat more artificial but also less compositionally diverse CL co-substrate and the specialized metabolic pathways necessary to metabolize such an unorthodox substrate. However, without transcriptomic and metabolomic data, which were not within the scope of this study, the identification and characterization of the metabolic properties of the few positively correlated OTUs remain to be addressed in future research.

Nucleotide BLAST revealed the OTUs positively correlated with the co-substrate addition associated with filamentous *Chloroflexi* often found in wastewater systems (OTU 13), which have not yet been cultured [64]. These *Prolixibacter*, which, in this framework, appear to be best described as r-strategist, are generally facultatively anaerobic, sugar-fermenting, and psychrotolerant rod-shaped bacteria [65]. They are found in a wide range of environments, for example, crude oil, tidal flats, or marine sediments, with some members of the genus known to be capable of nitrite reduction [65,66,67]. Another potential r-strategist [18] that was also positively correlated with co-substrate addition (OTU 12), although less pronounced, was assigned as a member of the genus *Proteiniphilum*—also frequently found in mesophilic biogas reactors or wastewater treatment facilities—and described as strictly anaerobic, proteolytic Gram-negative rods [68,69,70]. Sequences of both uncultured representatives are also often found in the available data on WWTP environmental sequencing efforts, and no similar changes in abundance were observed in the control reactors. While these two OTUs would be typical for an AD-reactor, this enrichment of already present but intrinsic organisms, that is, the species sorting of an existing community, appears to be the mechanism of selection in this case. A parallel emergence of an OTU in the CL co-substrate-fed reactors only, which was clearly PWASS-derived, was also observed and, in turn, suggests invasion under these changed environmental conditions [30].

This *Trichococcus* (OTU 27) has regularly been reported as a typical member of wastewater treatment sludge PWASS MCC [71], and is described as a Gram-positive filamentous bacterium with coccoid cells and that is able to utilize sugars, alcohols, and polysaccharides. Recent work furthermore suggests that *Trichococcus* may be capable of metabolizing glycerol [72], and a role in the metabolization of lecithin seems likely. In the control reactors, the full-scale WWTP or the FW experiment, the *Trichococcus* relative abundance rarely, and if so, barely, exceeded 0.2%; however, it was regularly observed with relative abundances in excess of 2% in the PWASS. The 10-fold increase in the relative abundance observed in the late experimental stages of the CL experiment could be indicative of invasion/recruitment from the PWASS after the formation of a suitable niche by the co-substrate addition. Yet, the selective pressure-connected loss of diversity could also lead to a loss of functional redundancy and, thus, a loss of resilience and, potentially, key suppliers of ecosystem services and stability in the long term [25,26,73].

## 5. Conclusions

In conclusion, the results show that microbial community organization in wastewater anaerobic co-digestion, with the mentioned co-substrates, is mainly governed by the inoculum-inhabiting core community. Stochastic effects and neutral theory define the framework of general suitability for the available niches, and a well-defined, rather small core community with little plasticity. In addition, the possibility for recruitment and invasion from the primary substrate was shown, following the formation of a new niche, while a simple rise in the substrate availability of substrates with comparable composition and complexity to the primary substrate had little effect on the microbial community. A way forward was recently outlined in great detail [30], and the conclusions put forward in this study contribute to—but should also be tested within—that framework. The dynamic thresholds defining which mechanism acts as a key driver should be identified. In addition, the testing applicability with different co-substrates or temperatures and the use of different -omics tools to further explore the observations made here should be investigated. An additional benefit of co-digestion is enhanced biogas formation within the expected margins, that is, a linear rise in biogas formation per added organic material with the high compositional complexity food waste co-substrate(s). The addition of highly artificial or specialized co-substrates, on the other hand, might lead to a loss of diversity and functional redundancy, and thus, could decrease the fitness of the system and have adverse downstream effects.

## Figures and Tables

**Table 1:** Characterization of the different substrates.

	PWASS ^a^	Food Waste (FW)	Canola Lecithin (CL)	WWTP ^b^
Total solids (g L^−1^)	33.3 ± 0.5	221.3	464.1	41.3 ± 10.9
Volatile solids (g L^−1^)	21.6 ± 0.4	197.7	463.4	23.8 ± 5.6
Raw protein ^c^	40.00%	15.68%	<0.65%	
Raw fat A/B ^c^	11.43%	18.21%	1.29% ^d,e^	
Raw fiber ^c^	5.71%	1.52%	1.08%	
ADL ^c^	5.71%	2.02%	<1.9%	
Carbohydrates ^f^	37.15%	62.57%	n.a.	
Total-N (mg L^−1^)	584.67	4210.0	271.0	
COD (g L^−1^)	31.23	256.75	529.0	

^a^ Average with standard deviation of 8 PWASS batches; individual concentrations of TS and VS with co-substrate can be seen in Table A1. ^b^ Sludge from the anaerobic digestors; average values according to personal communication with WWTP plant values taken between November 2019 and August 2020 (*n* = 74). ^c^ Values are given as percentage of the volatile solids fraction (VS). ^d^ Raw fat A + B. ^e^ Lecithin-specific phospholipids other than raw fat A/B (A + B) could not be determined. ^f^ Difference to 100%. n.a. = Not available.

## Data Availability

Sequencing data deposition—All nucleotide sequences obtained in this study were deposited in GenBank. Amplicon sequences from the 16S rRNA gene survey were deposited in the NCBI BioProject with the BioProject ID PRJNA745536.

## Supplementary Materials

The following are available online at https://www.mdpi.com/article/10.3390/bioengineering8120197/s1, Table S1: reads per sample and Domain, Table S2: Shannon Diversity.

## Abbreviations

The following abbreviations are used in this manuscript:

Co-AD	Anaerobic co-digestion
AD	Anaerobic digestion/-er
CL	Canola lecithin
FW	Food waste
OLR	Organic loading rate
ACC	Archaeal community composition
BCC	Bacterial community composition
MCC	Microbial community composition
OTU	Operational taxonomic unit
OOI	OTU of interest
PWASS	Primary (PS) and waste-activated sludge (WAS)
HRT/SRT	Hydraulic retention time/Solids retention time
IE	Inhabitant equivalents
WWTP	Wastewater treatment plant
TS	Total solids
VS	Volatile solids

## Appendix A

**Table A1:** Total solids (TS) and volatile solids (VS) of the used PWASS batches (1 to 8) during the two experiments (FW, CL) with the resulting organic loading rates of the daily feeding (OLR), with or without the respective co-substrate addition.

PWASS Experiment/Batch		TS	VS	Resulting OLR (kg VS m^−3^ d^−1^)			
		(g L^−1^)	(g L^−1^)				
				Control/0%	10%	20%	30%
FW	1	24.2	16.1	0.805			
	2	35.0	23.5	1.175	1.293		
	3	30.6	20.6	1.030	1.133	1.236	
	4	37.1	26.3	1.315			1.710
CL	5	37.5	27.6	1.380	1.518		
	6	33.4	22.6	1.130	1.243	1.356	
	7	36.8	22.3	1.115		1.338	1.450
	8	26.5	17.5	0.875			1.138

## References

[1] Appels L., Baeyens J., Degrève J., Dewil R. (2008). Principles and potential of the anaerobic digestion of waste-activated sludge. Prog. Energy Combust. Sci..

[2] Batstone D.J., Keller J., Angelidaki I., Kalyuzhnyi S.V., Pavlostathis S.G., Rozzi A., Sanders W.T., Siegrist H.A., Vavilin V.A. (2002). The IWA Anaerobic Digestion Model No 1 (ADM1). Water Sci. Technol..

[3] Ahn Y., Lee W., Kang S., Kim S.-H. (2019). Enhancement of Sewage Sludge Digestion by Co-digestion with Food Waste and Swine Waste. Waste Biomass-Valorization.

[4] Aichinger P., Wadhawan T., Kuprian M., Higgins M., Ebner C., Fimml C., Murthy S., Wett B. (2015). Synergistic co-digestion of solid-organic-waste and municipal-sewage-sludge: 1 plus 1 equals more than 2 in terms of biogas production and solids reduction. Water Res..

[5] Björn A., Yekta S.S., Ziels R.M., Gustafsson K., Svensson B.H., Karlsson A. (2017). Feasibility of OFMSW co-digestion with sewage sludge for increasing biogas production at wastewater treatment plants. Euro-Mediterr. J. Environ. Integr..

[6] Wickham R., Galway B., Bustamante H., Nghiem L.D. (2016). Biomethane potential evaluation of co-digestion of sewage sludge and organic wastes. Int. Biodeterior. Biodegrad..

[7] Mallapaty S. (2020). How China could be carbon neutral by mid-century. Nature.

[8] European Commission, Directorate General for Climate Action (2020). State of the Union 2020: EU Climate Target Plan 2030: Building a Modern, Sustainable and Resilient Europe.

[9] Long J.H., Aziz T.N., Reyes F.L.D.L., Ducoste J. (2012). Anaerobic co-digestion of fat, oil, and grease (FOG): A review of gas production and process limitations. Process. Saf. Environ. Prot..

[10] Keucken A., Habagil M., Batstone D., Jeppsson U., Arnell M. (2018). Anaerobic co-digestion of sludge and organic food waste-performance, inhibition, and impact on the microbial community. Energies.

[11] Czatzkowska M., Harnisz M., Korzeniewska E., Koniuszewska I. (2020). Inhibitors of the methane fermentation process with particular emphasis on the microbiological aspect: A review. Energy Sci. Eng..

[12] Zhao J., Liu Y., Wang D., Chen F., Li X., Zeng G., Yang Q. (2017). Potential impact of salinity on methane production from food waste anaerobic digestion. Waste Manag..

[13] Qu Y., Xiao C., Workie E., Zhang J., He Y., Tong Y.W. (2021). Bioelectrochemical Enhancement of Methanogenic Metabolism in Anaerobic Digestion of Food Waste Under Salt Stress Conditions. ACS Sustain. Chem. Eng..

[14] Misson G., Mainardis M., Marroni F., Peressotti A., Goi D. (2020). Environmental methane emissions from seagrass wrack and evaluation of salinity effect on microbial community composition. J. Clean. Prod..

[15] De Vrieze J., Christiaens M.E., Walraedt D., Devooght A., Ijaz U.Z., Boon N. (2017). Microbial community redundancy in anaerobic digestion drives process recovery after salinity exposure. Water Res..

[16] Kirkegaard R.H., McIlroy S.J., Kristensen J.M., Nierychlo M., Karst S.M., Dueholm M.S., Albertsen M., Nielsen P.H. (2017). Identifying the abundant and active microorganisms common to full-scale anaerobic digesters. Microbiology.

[17] Peces M., Astals S., Jensen P., Clarke W. (2018). Deterministic mechanisms define the long-term anaerobic digestion microbiome and its functionality regardless of the initial microbial community. Water Res..

[18] Wu L., Yang Y., Chen S., Shi Z.J., Zhao M., Zhu Z., Yang S., Qu Y., Ma Q., He Z. (2017). Microbial functional trait of rRNA operon copy numbers increases with organic levels in anaerobic digesters. ISME J..

[19] Calusinska M., Goux X., Fossépré M., Muller E., Wilmes P., Delfosse P. (2018). A year of monitoring 20 mesophilic full-scale bioreactors reveals the existence of stable but different core microbiomes in bio-waste and wastewater anaerobic digestion systems. Biotechnol. Biofuels.

[20] Wittebolle L., Marzorati M., Clement L., Balloi A., Daffonchio D., Heylen K., De Vos P., Verstraete W., Boon N. (2009). Initial community evenness favours functionality under selective stress. Nature.

[21] Allison S.D., Martiny J.B.H. (2008). Resistance, resilience, and redundancy in microbial communities. Proc. Natl. Acad. Sci. USA.

[22] Fernandez-Gonzalez N., Huber J.A., Vallino J.J. (2016). Microbial Communities Are Well Adapted to Disturbances in Energy Input. mSystems.

[23] Theuerl S., Klang J., Prochnow A. (2019). Process Disturbances in Agricultural Biogas Production—Causes, Mechanisms and Effects on the Biogas Microbiome: A Review. Energies.

[24] Regueiro L., Veiga P., Figueroa M., Alonso-Gutierrez J., Stams A., Lema J., Carballa M. (2012). Relationship between microbial activity and microbial community structure in six full-scale anaerobic digesters. Microbiol. Res..

[25] Langenheder S., Lindström E.S., Tranvik L.J. (2006). Structure and Function of Bacterial Communities Emerging from Different Sources under Identical Conditions. Appl. Environ. Microbiol..

[26] Peter H., Beier S., Bertilsson S., Lindström E., Langenheder S., Tranvik L.J. (2010). Function-specific response to depletion of microbial diversity. ISME J..

[27] De Vrieze J., Saunders A.M., He Y., Fang J., Nielsen P.H., Verstraete W., Boon N. (2015). Ammonia and temperature determine potential clustering in the anaerobic digestion microbiome. Water Res..

[28] Ju F., Lau F., Zhang T. (2017). Linking Microbial Community, Environmental Variables, and Methanogenesis in Anaerobic Biogas Digesters of Chemically Enhanced Primary Treatment Sludge. Environ. Sci. Technol..

[29] Kinnunen-Grubb M., Dechesne A., Proctor C., Hammes F., Johnson D., Quintela-Baluja M., Graham D., Daffonchio D., Fodelianakis S., Hahn N. (2016). A conceptual framework for invasion in microbial communities. ISME J..

[30] Fernandez-Gonzalez N., Braz G.H.R., Regueiro L., Lema J.M., Carballa M. (2020). Microbial invasions in sludge anaerobic digesters. Appl. Microbiol. Biotechnol..

[31] Brown B.L., Barney J.N. (2021). Rethinking Biological Invasions as a Metacommunity Problem. Front. Ecol. Evol..

[32] Uhlenhut F., Schlüter K., Gallert C. (2018). Wet biowaste digestion: ADM1 model improvement by implementation of known genera and activity of propionate oxidizing bacteria. Water Res..

[33] (2018). Standard Methods for the Examination of Water and Wastewater.

[34] Takahashi S., Tomita J., Nishioka K., Hisada T., Nishijima M. (2014). Development of a Prokaryotic Universal Primer for Simultaneous Analysis of Bacteria and Archaea Using Next-Generation Sequencing. PLoS ONE.

[35] Dyksma S., Jansen L., Gallert C. (2020). Syntrophic acetate oxidation replaces acetoclastic methanogenesis during thermophilic digestion of biowaste. Microbiome.

[36] Deutsche Vereinigung für Wasserwirtschaft A und A (2020). Merkblatt DWA-Co-Vergärung in Kommunalen Klärschlammfaulbehältern, Abfallvergärungsanlagen und Landwirtschaftlichen Biogasanlagen.

[37] Dyksma S., Gallert C. (2019). Candidatus Syntrophosphaera thermopropionivorans: A novel player in syntrophic propionate oxidation during anaerobic digestion. Environ. Microbiol. Rep..

[38] Astals S., Esteban-Gutiérrez M., Arevalo T.F., Aymerich E., García-Heras J., Mata-Alvarez J. (2013). Anaerobic digestion of seven different sewage sludges: A biodegradability and modelling study. Water Res..

[39] Li J., Wei L., Duan Q., Hu G., Zhang G. (2014). Semi-continuous anaerobic co-digestion of dairy manure with three crop residues for biogas production. Bioresour. Technol..

[40] Zhang C., Su H., Wang Z., Tan T., Qin P. (2015). Biogas by Semi-Continuous Anaerobic Digestion of Food Waste. Appl. Biochem. Biotechnol..

[41] Weiterbildender Studiengang Wasser und Umwelt, Deutsche Vereinigung für Wasserwirtschaft, Abwasser und Abfall (2009). Abwasserbehandlung: Gewässerbelastung, Bemessungsgrundlagen, Mechanische Verfahren, Biologische Verfahren, Reststoffe aus der Abwasserbehandlung, Kleinkläranlagen.

[42] Nasir I.M., Ghazi T.I., Omar R., Idris A. (2013). Batch and semi-continuous biogas production from cattle manure. Int. J. Eng. Technol..

[43] Lovley D.R., Phillips E.J. (1987). Competitive mechanisms for inhibition of sulfate reduction and methane production in the zone of ferric iron reduction in sediments. Appl. Environ. Microbiol..

[44] Walczak M., Cywinska A. (2007). Application of selected chemical compounds to limit the growth of filamentous bacteria in activated sludge. Environ. Prot. Eng..

[45] Adams H., Crump B., Kling G. (2014). Metacommunity dynamics of bacteria in an arctic lake: The impact of species sorting and mass effects on bacterial production and biogeography. Front. Microbiol..

[46] Li L., He Q., Ma Y., Wang X., Peng X. (2015). Dynamics of microbial community in a mesophilic anaerobic digester treating food waste: Relationship between community structure and process stability. Bioresour. Technol..

[47] Lucas R., Kuchenbuch A., Fetzer I., Harms H., Kleinsteuber S. (2015). Long-term monitoring reveals stable and remarkably similar microbial communities in parallel full-scale biogas reactors digesting energy crops. FEMS Microbiol. Ecol..

[48] Orellana E., Davies-Sala C., Guerrero L.D., Vardé I., Altina M., Lorenzo M.C., Figuerola E.L.M., Pontiggia R.M., Erijman L. (2019). Microbiome network analysis of co-occurrence patterns in anaerobic co-digestion of sewage sludge and food waste. Water Sci. Technol..

[49] Sundberg C., Abu Al-Soud W., Larsson M., Alm E., Yekta S.S., Svensson B.H., Sørensen S., Karlsson A. (2013). 454 pyrosequencing analyses of bacterial and archaeal richness in 21 full-scale biogas digesters. FEMS Microbiol. Ecol..

[50] Abendroth C., Vilanova C., Günther T., Luschnig O., Porcar M. (2015). Eubacteria and archaea communities in seven mesophile anaerobic digester plants in Germany. Biotechnol. Biofuels.

[51] Shade A., Peter H., Allison S.D., Baho D.L., Berga M., Buergmann H., Huber D.H., Langenheder S., Lennon J.T., Martiny J.B.H. (2012). Fundamentals of Microbial Community Resistance and Resilience. Front. Microbiol..

[52] Lam T.Y., Mei R., Wu Z., Lee P.K., Liu W.-T., Lee P.-H. (2020). Superior resolution characterisation of microbial diversity in anaerobic digesters using full-length 16S rRNA gene amplicon sequencing. Water Res..

[53] Fenchel T., King G.M., Blackburn T.H. (2012). Bacterial Metabolism. Bacterial Biogeochemistry.

[54] Wormald R., Humphreys P. (2019). Hydrogenotrophic methanogenesis dominates at high pH. Access Microbiol..

[55] Hunik J.H., Hamelers H.V.M., Koster I.W. (1990). Growth-rate inhibition of acetoclastic methanogens by ammonia and pH in poultry manure digestion. Biol. Wastes.

[56] Wormald R.M., Rout S.P., Mayes W., Gomes H., Humphreys P.N. (2020). Hydrogenotrophic Methanogenesis Under Alkaline Conditions. Front. Microbiol..

[57] Kotsyurbenko O.R., Friedrich M.W., Simankova M.V., Nozhevnikova A.N., Golyshin P.N., Timmis K.N., Conrad R. (2007). Shift from Acetoclastic to H 2 -Dependent Methanogenesis in a West Siberian Peat Bog at Low pH Values and Isolation of an Acidophilic Methanobacterium Strain. Appl. Environ. Microbiol..

[58] Li L., Ma Z.S. (2020). Species Sorting and Neutral Theory Analyses Reveal Archaeal and Bacterial Communities are Assembled Differently in Hot Springs. Front. Bioeng. Biotechnol..

[59] Berga M., Székely A.J., Langenheder S. (2012). Effects of disturbance intensity and frequency on bacterial community composition and function. PLoS ONE.

[60] Lee J.E., Buckley H.L., Etienne R.S., Lear G. (2013). Both species sorting and neutral processes drive assembly of bacterial communities in aquatic microcosms. FEMS Microbiol. Ecol..

[61] Leng L., Yang P., Singh S., Zhuang H., Xu L., Chen W.-H., Dolfing J., Li D., Zhang Y., Zeng H. (2018). A review on the bioenergetics of anaerobic microbial metabolism close to the thermodynamic limits and its implications for digestion applications. Bioresour. Technol..

[62] Zhang W., Xu Y., Dong B., Dai X. (2019). Characterizing the sludge moisture distribution during anaerobic digestion process through various approaches. Sci. Total. Environ..

[63] Puig-Castellví F., Cardona L., Bouveresse D.J.-R., Cordella C.B.Y., Mazéas L., Rutledge D.N., Chapleur O. (2020). Assessment of the microbial interplay during anaerobic co-digestion of wastewater sludge using common components analysis. PLoS ONE.

[64] Björnsson L., Hugenholtz P., Tyson G.W., Blackall L.L. (2002). Filamentous Chloroflexi (green non-sulfur bacteria) are abundant in wastewater treatment processes with biological nutrient removalccThe EMBL accession numbers for the sequences reported in this paper are X84472 (strain SBR1029 16S rDNA), X84474 (strain SBR1031 16S rDNA), X84498 (strain SBR1064 16S rDNA), X84565 (strain SBR2022 16S rDNA), X84576 (strain SBR2037 16S rDNA) and X84607 (strain SBR2076 16S rDNA). Microbiology.

[65] Holmes D.E., Nevin K.P., Woodard T.L., Peacock A.D., Lovley D.R. (2007). Prolixibacter bellariivorans gen. nov., sp. nov., a sugar-fermenting, psychrotolerant anaerobe of the phylum Bacteroidetes, isolated from a marine-sediment fuel cell. Int. J. Syst. Evol. Microbiol..

[66] Iino T., Sakamoto M., Ohkuma M. (2015). Prolixibacter denitrificans sp. nov., an iron-corroding, facultatively aerobic, nitrate-reducing bacterium isolated from crude oil, and emended descriptions of the genus Prolixibacter and Prolixibacter bellariivorans. Int. J. Syst. Evol. Microbiol..

[67] Iino T., Mori K., Itoh T., Kudo T., Suzuki K.-I., Ohkuma M. (2014). Description of Mariniphaga anaerophila gen. nov., sp. nov., a facultatively aerobic marine bacterium isolated from tidal flat sediment, reclassification of the Draconibacteriaceae as a later heterotypic synonym of the Prolixibacteraceae and description of the family Marinifilaceae fam. nov. Int. J. Syst. Evol. Microbiol..

[68] Chen S., Dong X. (2005). Proteiniphilum acetatigenes gen. nov., sp. nov., from a UASB reactor treating brewery wastewater. Int. J. Syst. Evol. Microbiol..

[69] Hahnke S., Langer T., Koeck D.E., Klocke M. (2016). Description of Proteiniphilum saccharofermentans sp. nov., Petrimonas mucosa sp. nov. and Fermentimonas caenicola gen. nov., sp. nov., isolated from mesophilic laboratory-scale biogas reactors, and emended description of the genus Proteiniphilum. Int. J. Syst. Evol. Microbiol..

[70] Whitman W.B., Parte A.C. (2009). Bergey’s Manual of Systematic Bacteriology.

[71] Scheff G., Salcher O., Lingens F. (1984). Trichococcus flocculiformis gen. nov. sp. nov. A new gram-positive filamentous bacterium isolated from bulking sludge. Appl. Microbiol. Biotechnol..

[72] Strepis N., Naranjo H.D., Meier-Kolthoff J., Göker M., Shapiro N., Kyrpides N., Klenk H.-P., Schaap P.J., Stams A.J.M., Sousa D.Z. (2020). Genome-guided analysis allows the identification of novel physiological traits in Trichococcus species. BMC Genom..

[73] Cydzik-Kwiatkowska A., Zielińska M. (2016). Bacterial communities in full-scale wastewater treatment systems. World J. Microbiol. Biotechnol..

