# Supplementary material for: Mechanisms Driving Microbial Community Composition in Anaerobic Co-Digestion of Waste-Activated Sewage Sludge
Source: Bioengineering (Basel). 2021 Nov 30;8(12):197. doi: 10.3390/bioengineering8120197 (PMC8699016; doi:10.3390/bioengineering8120197)
Supplement: Supplementary file 1 [file bioengineering-08-00197-s001.zip › bioengineering-1482164-supplementary.pdf]

Supplementary

Table S1 reads per sample and Domain, not including Eukaryote sequences

Sample	reads total	reads Archaea	reads Bacteria
Inoculum FW 00 FT	280112	8870	280112
Control FW 01 IV	201339	19039	201339
Control FW 01 V	288360	29026	288360
Control FW 01 VI	152812	12259	152812
Control FW 02 IV	263303	19594	263303
Control FW 02 V	65749	5590	65749
Control FW 02 VI	105938	8896	105938
Control FW 03 IV	249726	22569	249726
Control FW 03 V	231245	15856	231245
Control FW 03 VI	261460	22382	261460
Control FW 04 IV	276280	13762	276280
Control FW 04 V	293577	15741	293577
Control FW 04 VI	296935	18210	296935
Experiment FW 01 I	149585	10937	149585
Experiment FW 01 II	163115	15715	163115
Experiment FW 01 III	243854	23620	243854
Experiment FW 02 I	111211	7025	111211
Experiment FW 02 II	178783	13330	178783
Experiment FW 02 III	168336	11136	168336
Experiment FW 03 I	185405	15158	185405
Experiment FW 03 II	270371	19566	270371
Experiment FW 03 III	223584	15877	223584
Experiment FW 04 I	74361	4898	74361
Experiment FW 04 II	65274	4293	65274
Experiment FW 04 III	298996	18549	298996
Inoculum CL 00 FT	192730	12306	192730
Control CL 01 IV	82742	3502	82742
Control CL 01 V	79589	2927	79589
Control CL 01 VI	98324	4139	98324
Control CL 02 IV	85216	4575	85216
Control CL 02 V	102945	4235	102945
Control CL 02 VI	108543	5904	108543
Control CL 03 IV	74002	2913	74002
Control CL 03 V	94796	2900	94796
Control CL 03 VI	83768	2705	83768
Control CL 04 IV	79766	3923	79766
Control CL 04 V	67265	3685	67265
Control CL 04 VI	86172	3835	86172
Experiment CL 01 I	86495	3056	86495
Experiment CL 01 II	44479	2162	44479
Experiment CL 01 III	84494	3755	84494
Experiment CL 02 I	144233	6078	144233
Experiment CL 02 II	88119	3842	88119
Experiment CL 02 III	108729	4469	108729
Experiment CL 03 I	114169	3327	114169
Experiment CL 03 II	87545	2311	87545
Experiment CL 03 III	162026	4728	162026
Experiment CL 04 I	74796	2919	74796

Experiment CL 04 II	77748	3110	77748
Experiment CL 04 III	99390	4110	99390
PWASS CL 01 All	61674	86	61674
PWASS CL 02 All	204026	11764	204026
PWASS CL 03 All	169445	723	169445
PWASS CL 04 All	78244	248	78244
PWASS FW 01 All	97649	777	97649
PWASS FW 02 All	233823	939	233823
PWASS FW 03 All	59138	164	59138
PWASS FW 04 All	18630	51	18630
WWTP CL 02 FT	231875	17870	231875
WWTP CL 03 FT	254850	14953	254850
WWTP CL 04 FT	251352	18986	251352
WWTP FW 01 FT	340821	20850	340821
WWTP FW 02 FT	372229	16087	372229
WWTP FW 03 FT	392501	24419	392501
WWTP FW 04 FT	210700	21230	210700

Table S2. Shannon Diversity.

Treatment	Experiment	runtime in days	Shannon index
Inoculum T0	FW	0	5,19
Control T1	FW	27	5,05
Control T2	FW	41	5,10
Control T3	FW	90	5,02
Control T4	FW	125	4,98
Experiment T1 (0%)	FW	27	5,07
Experiment T2 (10%)	FW	41	5,04
Experiment T3 (20%)	FW	90	5,01
Experiment T4 (30%)	FW	125	4,95
Inoculum T0	CL	0	4,97
Control T1	CL	1	5,01
Control T2	CL	29	4,98
Control T3	CL	71	5,15
Control T4	CL	92	5,13
Experiment T1 (0%)	CL	1	5,02
Experiment T2 (10%)	CL	29	4,88
Experiment T3 (20%)	CL	71	4,78
Experiment T4 (30%)	CL	92	4,59
PWASS 1	FW	0	6,27
PWASS 2	FW	9	6,54
PWASS 3	FW	51	6,02
PWASS 4	FW	95	5,94
PWASS 5	CL	2	5,64
PWASS 6	CL	31	4,98
PWASS 7	CL	64	6,15
PWASS 8	CL	93	5,53
WWTP FW T1	FW	14	5,22
WWTP FW T2	FW	35	4,75
WWTP FW T3	FW	77	5,47
WWTP FW T4	FW	113	5,26
WWTP CL T2	CL	43	5,09

WWTP CL T3	CL	70	5,04
WWTP CL T4	CL	84	4,91

Shown are different Treatments of the two experiments (FW = Food Waste, CL = Canola Lecithin) at different timepoints from inoculation (T0) to addition of 30% co-substrate in the experimental reactors (T4), with the corresponding runtime and respective PWASS batches and full scale WWTP samples (WWTP)
